# Dietary fiber and the glycemic index: a background paper for the Nordic Nutrition Recommendations 2012

**DOI:** 10.3402/fnr.v57i0.20709

**Published:** 2013-03-25

**Authors:** Nina Cecilie Øverby, Emily Sonestedt, David E. Laaksonen, Bryndis Eva Birgisdottir

**Affiliations:** 1 Department of Public Health, Sport and Nutrition, University of Agder, Kristiansand, Norway; 2 Department of Clinical Sciences – Malmö, Lund University, Malmö, Sweden; 3 Department of Medicine, Kuopio University Hospital, Kuopio, Finland; 4 Unit for Nutrition Research, Landspitali-University Hospital and University of Iceland, Reykjavik, Iceland

**Keywords:** dietary fiber, glycemic index, Nordic Nutrition Recommendations

## Abstract

The aim of this study is to review recent data on dietary fiber (DF) and the glycemic index (GI), with special focus on studies from the Nordic countries regarding cardiometabolic risk factors, type 2 diabetes, cardiovascular disease, cancer, and total mortality. In this study, recent guidelines and scientific background papers or updates on older reports on DF and GI published between 2000 and 2011 from the US, EU, WHO, and the World Cancer Research Fund were reviewed, as well as prospective cohort and intervention studies carried out in the Nordic countries. All of the reports support the role for fiber-rich foods and DF as an important part of a healthy diet. All of the five identified Nordic papers found protective associations between high intake of DF and health outcomes; lower risk of cardiovascular disease, type 2 diabetes, colorectal and breast cancer. None of the reports and few of the Nordic papers found clear evidence for the GI in prevention of risk factors or diseases in healthy populations, although association was found in sub-groups, e.g. overweight and obese individuals and suggestive for prevention of type 2 diabetes. It was concluded that DF is associated with decreased risk of different chronic diseases and metabolic conditions. There is not enough evidence that choosing foods with low GI will decrease the risk of chronic diseases in the population overall. However, there is suggestive evidence that ranking food based on their GI might be of use for overweight and obese individuals. Issues regarding methodology, validity and practicality of the GI remain to be clarified.

The present literature review is a part of the background documentation for the 5th edition of the Nordic Nutrition Recommendations (NNR) 2012 with the aim of reviewing and updating the scientific basis of the 4th edition of the NNR issued in 2004 (1). The NNR 2012 project focuses mainly on a revision of those areas in which new scientific knowledge has emerged since the 4th edition, with special relevance for the Nordic setting. A number of systematic literature reviews form the basis for establishment of dietary reference values in the 5th edition of NNR.

The task of the present expert group was to systematically review studies regarding carbohydrate quantity and quality in association with health outcomes. A systematic review on sugar and cardiometabolic risk factors, type 2 diabetes, cardiovascular disease and total mortality has been published previously (2). The aim of the present paper was to review and summarize recent advances in studies on dietary fiber (DF) and the glycemic index (GI) with special focus on Nordic countries. The recommendation for DF intake in the NNR 2004 is 25–35 g/d or 3 g/MJ (1). In the carbohydrate chapter (1), the state of the art of GI is included but no recommendation is given. We decided to review official updated guidelines and recently published scientific background papers on DF and the GI from the United States (US) (3–5), European Food Safety Authority (EFSA) (6), Food and Agricultural Organization/World Health Organization (WHO) (7) and The World Cancer Research Fund report (8). Additionally, a systematic literature search was made for studies in the Nordic population published 2000–2011 on DF and the GI with cardiometabolic risk factors, type 2 diabetes, cardiovascular disease, total mortality and cancer as endpoints.

## Methods

### Guidelines and reports

The group evaluated the Dietary Guidelines from the United States Department of Agriculture (USDA) (3–5) and the Dietary Reference Values from EFSA (6), both published in 2010. Furthermore, an updated report on Carbohydrates in Human Nutrition from WHO published 2007 (7), a report from The World Cancer Research Fund (8), and a review on the GI from the Nordic Council of Ministers from 2005 (9) was included in the review. When reporting conclusions from these reports, same or similar wording is used.

### Nordic studies

For the original articles from the Nordic countries, we defined the literature search and criteria for inclusion and exclusion, set prior to abstract screening. The eligibility criteria is similar to the criteria used in the systematic review on sugar and cardiometabolic risk factors by the same authors (2) and were based on the following aspects:

#### Exposure/intervention

We included studies examining DF, the GI and glycemic load (GL) as indicator of exposure. The GL is calculated by multiplying the GI of a food item with the amount of available carbohydrates (g) in a portion of the food. To identify studies from the Nordic countries, we only included studies with the words Scandinavian, Nordic, Iceland, Norway, Denmark, Sweden or Finland in title or abstract (Appendix 1).

#### Study design

Prospective observational studies (cohort or nested case-control) with a length of follow-up of four years or more, or randomized and controlled interventions of at least 4 weeks were included. For randomized studies, the drop-out rate had to be less than 50%. Studies including more than one intervention in the experimental arm were not included.

#### Outcome

We included cardiovascular disease, type 2 diabetes, cancer, and all-cause mortality as outcome measures. Glucose tolerance, insulin sensitivity, blood lipids, inflammation markers, and blood pressure were chosen as intermediate markers.

#### Control

For intervention trials with GI, the control diet had to include replacement of low GI food with food with similar contribution from macronutrients and similar DF content when relevant. For intervention trials with DF, the replacement food had to include similar contribution from other macronutrients. Studies without a control group were not considered.

#### Population

The population was defined as the general healthy population including all age groups. We also considered studies that included individuals that were overweight.

#### Language

English or a Nordic language.

#### Article type

Original articles and systematic reviews.

#### Time period

From January 2000 until December 2011.

The literature search was performed in February 2012 in collaboration with a librarian in order to ensure objectivity. Search terms are presented in Appendix I (DF) and Appendix II (GI and GL). The search included papers found in Medline through the PubMed platform, supplied by United States National Library of Medicine (http://www.ncbi.nlm.nih.gov/pubmed).

After receiving the list of abstracts from the search, one expert reviewed and ordered the articles of interest in full text. Abstracts not relevant for the research questions were excluded, and no reason was recorded for exclusion. The full text papers were reviewed by one expert. No further quality assessment and grading of evidence were performed for the papers included in this review. The current paper is therefore not a systematic review but depends on the objectivity of other researchers making reports and opinions for international organizations and bodies.

## Results

### Dietary fiber

#### Definitions

There are two main approaches to the definition of DF: *the plant-rich diet approach* and the *indigestibility approach*. The FAO/WHO Scientific update on carbohydrates in human nutrition clearly states that the plant-rich diet approach is the most suitable in a public health perspective (10). This approach specifically targets fruits, vegetables and whole grain products that are consistently linked with health benefits. In the indigestibility definition, the term ‘DF’ is not completely well-defined as it is not always linked to the chemical structure. The Codex Alimentarius Commission defined DF as carbohydrate polymers (also including other quantitatively minor components (mainly lignins) that are associated with DF-polysaccharides in the plant wall) with ten or more monomeric units, which are not hydrolyzed by the endogenous enzymes in the small intestine of humans (11). This definition included both carbohydrates naturally occurring in the food, and carbohydrates obtained from foods and synthetic carbohydrates shown to have positive health effects. Whether Carbohydrates with 3–9 monomers are DF or not should according to the Codex be decided by national authorities However, there is strong consensus in the scientific community that there is no scientific basis of the cut point of 10 or more monomers (11).

The substances contained in the concept of DF, are by definition, resistant to hydrolysis and absorption in the small intestine. They are passed on down to the large intestine unmodified and are more or less fermented by the intestinal bacteria. The different types of DF have different physiological properties and potential effects on health. DF can affect digestion and absorption in the upper and lower gastrointestinal tract but also the levels of blood glucose, insulin, blood lipids and cholesterol, satiety and energy balance, and composition of intestinal micro flora and its degradation products (1). Another expert group within the NNR project has reviewed the literature on whole grain and health (12).

#### Dietary guidelines for Americans 2010

In the Report of the Dietary Guidelines Advisory Committee on the Dietary Guidelines for Americans, 2010 (13), the chapter on carbohydrates was partly based on systematic reviews (13–16). For DF there was only one scientific question asked by the Committee: Is intake of DF related to adiposity in children? In addition, two questions on whole grain intake (including DF) were asked. The questions asked and the conclusions of the systematic literature reviews are found in Table 1, (13). This resulted in the following guideline regarding DF and whole grains: ‘Americans should choose fiber-rich carbohydrate foods such as whole grains, vegetables, fruits, and cooked dry beans and peas as staples in the diet’ (13). In addition, dietary reference intake for DF is from 19 g/d in children to 38 g/d in male adults (14).

**Table 1:** Questions asked and the conclusions of the systematic literature reviews for the dietary guidelines for Americans 2010 regarding DF

Question	Conclusion	Number of studies
Is intake of dietary fiber related to adiposity in children? (15)	‘There is insufficient evidence that dietary fiber is associated with adiposity in children.’	Based on 2 RCTs and 2 prospective cohorts, 1 cohort and 1 cross sectional study
What is the relationship between whole grain intake and cardiovascular disease? (16)	‘A moderate body of evidence shows that whole grain intake, which includes cereal fiber, protects against cardiovascular disease’	Based on one systematic review, two meta-analyses, one randomized controlled trial, 3 prospective cohorts that were published after the systematic review
What is the relationship between whole grain intake and body weight? (17)	‘Moderate evidence shows that intake of whole grains and grain fiber is associated with lower body weight’	Based on one systematic review, one systematic review/meta-analyses, 2 RCTs, 3 cross sectional studies

#### The Scientific Opinion of the European Food Safety Authority

According to the scientific opinion on dietary reference values on carbohydrates and DF by the EFSA (6), the most suitable criterion for establishing an adequate intake of DF is its role in bowel function. The panel considered DF intakes of 25 g per day to be adequate for normal laxation in adults. Although there is limited evidence to set adequate intakes for children, a DF intake of 2 g/MJ is considered adequate for normal laxation in children from 1 year (6). The EFSA Scientific Opinion used the indigestibility approach for their DF definition.

Based on the literature review, EFSA concludes that increasing intakes of foods rich in DF are associated with reduced risk of impaired glucose control. Favorable effects of DF were observed at>2.6 g/MJ; about 30 g per day. However, they state that the contribution of DF per se to this effect remains to be established (6). In relation to serum lipids, the EFSA opinion refers to a meta-analysis and recent research that viscous types of DF may contribute to reducing total and LDL-cholesterol concentrations, but the effects are limited at amounts usually consumed from foods (6). The EFSA also concludes that DF is associated with lower blood pressure, body weight, and risk of colorectal cancer and type 2 diabetes. In relation to cardiovascular disease, they refer to a meta-analysis of 10 prospective cohorts to conclude that an increase in 10 g DF per day was associated with 14% lower risk of all coronary events (6, 18).

#### FAO/WHO scientific update on carbohydrates in human nutrition 2007

The FAO/WHO scientific update on carbohydrates and health presents several reviews (not systematic). The conclusions from two of these are presented below: carbohydrates and cardiovascular disease and disorders of carbohydrate metabolism (19), and carbohydrates and cancer (20). The conclusions on DF intake are presented in Table 2. FAO/WHO use the plant-rich diet approach for the definition of DF.

**Table 2:** Conclusions on DF in relation to cardiovascular disease and cancer in the scientific update from FAO/WHO

Disease	Conclusion
Cardiovascular disease (19)	‘Whole grains, legumes, vegetables and intact fruits are the most appropriate sources of carbohydrates. There is strong evidence that they are associated with reduced risk of cardiovascular disease. These carbohydrate-containing foods are rich sources of DF (defined as non-starch polysaccharides), which protects against type 2 diabetes and other cardiovascular risk factors. However, to date there is no good evidence of protection against cardiovascular disease and diabetes when various oligosaccharides or polysaccharides or other isolated components of whole grains, fruits, vegetables and legumes are added to functional and manufactured food.’
Cancer (20)	‘There is a moderately large amount of data on the possible association between DF and the risk of colorectal cancer; the results suggest that DF may reduce the risk for colorectal cancer.’

The FAO/WHO Scientific Update conclude that the recommendations of the 2002 WHO/FAO Expert Consultation are compatible with the outcomes of the Scientific Update 2007 with some caveats (7). Precise amounts of DF as non-starch polysaccharide were not recommended by 2002 WHO/FAO Expert Consultation. It was considered that the recommended intakes of fruit, vegetables, legumes and regular consumption of whole grain cereals would provide adequate intakes of total DF (7), which is further stressed in the 2007 update (see Table 2).

#### Recommendations by the World Cancer Research Fund and the American Institute for Cancer Research

The updated meta-analysis from the World Cancer Research Fund published in 2011 includes 15 prospective cohort studies compared with eight studies in the previous Expert Report from 2007. The updated meta-analysis shows more consistent results regarding colorectal cancer and show a 10% decreased risk per 10 g increase in DF intake per day. The evidence that foods containing DF protect against colorectal cancer was therefore upgraded from probable to convincing by the Expert Panel (21).

#### Review of studies on dietary fiber from the Nordic countries published in 2000–2011

From the 128 identified abstracts, five relevant studies were included. Only studies examining total DF intake were included, i.e. studies focusing on single types of DF such as β-glucans were not included.

The included studies are summarized in Table 3. Three of the five studies examined the relation between DF intake and different types of cancers (22–24), while the other two studies examined the relation between DF intake and stroke and type 2 diabetes, respectively (25, 26).

**Table 3:** Nordic studies on dietary fiber intake and different metabolic conditions and diseases

	Outcome	*n*	Sex	Age	Follow up	Adjustments	Results
Hansen, L., Skeie G., et al. (22)	Colon and rectal cancer	Among 108,081 cohort members 1,168 incident cases (691 colon, 477 rectal cancer) were diagnosed	M/F		11.3 years (median)	BMI, education, smoking status, hormone replacement therapy use (for women) and intake of alcohol and red and processed meat.	Incidence rate ratio=0.94 (95% CI: 0.91, 0.98) in men and 0.97 (0.93, 1.00) in women for increased intake of 2 g cereal DF per day.
Larsson, S. C., Mannisto S., et al. (25)	Stroke	Among 26,556 Finnish male smokers, 2,702 cerebral infarctions, 383 intracerebral hemorrhages and 196 subarachnoid hemorrhages were ascertained	M	50–69	13.6 years (mean)	Age, supplementation group, number of cigarettes smoked daily, BMI, systolic and diastolic blood pressures, serum total cholesterol, serum high-HDL, history of diabetes and coronary heart disease, leisure-time physical activity, and intakes of alcohol, total energy, folate and magnesium.	Relative risk of cerebral infarction=0.86 (95% CI: 0.76, 0.99) for the highest vs. lowest quintile vegetable DF.
Mattisson, I., Wirfalt E., et al. (20)	Postmenopausal Breast cancer	Among 11 726 postmenopausal women, 342 incident cases were diagnosed	F	50 years or older	During 89 602 person-years of follow-up	Diet interviewer, season of diet interview, method version, age, change of dietary habits, total energy, current hormone use, age at first child, height, waist, leisure time physical activity, age at menarche, educational level.	Incidence rate ratio 0.58 (95% CI: 0.40, 0.84) for the highest vs. lowest quintile of DF intake.
Montonen, J., Knekt P., et al. (26)	Type 2 diabetes	Among 2,286 men and 2,030 women, 54 men and 102 women were diagnosed	M/F	40–69 years	10 years	Adjusted for age, sex, geographic area, smoking, BMI, and intakes of energy, fruit and berries, and vegetables.	Relative risk of type 2 diabetes=0.39 (95% CI: 0.20, 0.77). for the highest vs. lowest quartiles of cereal DF intake.
Suzuki, R., Rylander-Rudqvist T., et al. (24)	Breast cancer	Among 51,823 postmenopausal women, 1,188 breast cancer cases with known ER/PR status were diagnosed.	F	>57.9	8.3 years	Adjusted for age, height, body mass index, education, parity, age at first birth, age at menarche, age at menopause, type of menopause, use of oral contraceptives, use of postmenopausal hormones, family history of breast cancer among first-degree relatives, history of benign breast disease, total energy intake, energy-adjusted total fat intake, intake of fruits and vegetables and alcohol intake.	When comparing the highest to the lowest quintile, a non-significant inverse associations between total fiber intake and the risk of all tumor subtypes was observed; the Relative risk=0.85 (95% CI: 0.69, 1.05) for overall, 0.85 (0.64, 1.13) for estrogen receptor (ER)/progesterone receptor (PR)- breast cancer, 0.83 (0.52, 1.31) for ER+PR- and 0.94 (0.49, 1.80) for ER-PR-for highest vs.- lowest quintile of total DF. For specific fiber, the researchers observed statistically significant risk reductions for overall (34%) and for ER+PR+ (38%) for the highest versus lowest quintile of fruit fiber, and non-significant inverse associations for other subtypes of cancer and types of fiber.

M/F: male/female

Hansen et al. found a protective role of total and cereal DF intake in the development of colon cancer in the HELGA cohort. This cohort included three prospective Scandinavian cohorts with 1,168 incident cases among about 108,000 cohort members (22). Mattison et al. showed that high DF intakes were associated with a lower risk of postmenopausal breast cancer in the Malmö Diet and Cancer Cohort including 11, 726 women (23). Suzuki et al. found no significant associations between total DF intake and the risk of breast cancer in the Swedish Mammography Screening Cohort including almost 52,000 women (24). However, for fruit fiber, they found a statistically significant risk reduction for breast cancer for the highest versus lowest quintile.

Results from the Finnish Mobile Clinic Health Examination Survey, showed a significant inverse association between DF intake and risk of type 2 diabetes (26). In total 2,286 men and 2,030 women aged 40–69 and initially free of diabetes were included and followed for 10 years. The relative risk of type 2 diabetes between the extreme quartiles of cereal DF was 0.39 (see table 3).

Larsson et al. found no significant association between intake of total DF, water-soluble DF, water-insoluble DF, or DF derived from fruit or cereal sources and risk of any stroke subtype within the Alpha-Tocopherol, Beta-Carotene Cancer Prevention Study including 26,556 male smokers (25). However, vegetable DF was inversely associated with the risk of cerebral infarction (Table 3).

## Glycemic index

### 

#### Definitions

Postprandial glycemia refers to the elevation of blood glucose concentrations after consumption of a food or a meal, and is a normal physiological response which varies in magnitude and duration. The concept of GI lists food by its proposed effect on postprandial blood glucose compared to a reference food, or more recently, to pure glucose. GI can be influenced by the chemical and physical nature of the food or meal consumed but also by individual factors (27). These factors include nutrients such as certain types of DF, starch structure, type of sugar, fat and protein content, water, cellular structure and other structure related factors, molecular interactions, particle size distribution, presence of amylase inhibitors or organic acids, method of food preparation and degree of chewing (9).

The concept of the GI was developed in 1981, mainly in relation to people with diabetes (28). Current guidelines for the treatment of people with diabetes include the GI as a helpful additional indicator regarding the appropriate carbohydrate containing foods for inclusion in the diet (29, 30). Studies have also examined whether choosing food according to its GI could prevent the development of different diseases or metabolic conditions. The results are not consistent and methodological challenges have been pointed out as a potential explanation.

First, considerable individual differences in glucose responses can be found when testing various food items. Current guidelines on the number of persons necessary for testing a food item have been debated (27) as well as age and training state of subjects (31, 32). Second, GI is measured using 50 g of available carbohydrates from both the test food and the reference food (glucose). However, the availability of carbohydrates in some food items (for example due to resistant starch) may be overestimated resulting in lower amount of carbohydrates absorbed that might partly explain lower GI (33). This should however be over-come by analyzing the available carbohydrate portion. The glycemic index should only be used to rank food items with at least 10–20 g of available carbohydrates in a portion of the food (9).

Third, many cohort studies do not measure the GI of food in their country, but instead rely on international table values, which may not be suitable for local food items (34) and the comprehensiveness of the dietary questionnaires in epidemiological studies varies (27). Fourth, controlling for the effects of different types of food, DF and other macronutrients is difficult in both epidemiological studies and randomized controlled trials, and in many trials, low-GI and control diets generally differ in other potentially confounding aspects, such as soluble and insoluble fiber content, or viscosity of fiber and other macronutrient content. It can be hard to disentangle the positive effect due to lower glycemic and insulinemic response from positive effect due to other qualities of the food (27, 33). Studies on fiber have similar challenges.

Fifth, the GI does not take onto account the ‘second meal effect’ (the effect of a previous meal on the postprandial glycemia of a second meal) and meal frequency, which are important factors influencing the glycemic response (1). These can be controlled for in intervention studies but can rarely be accounted for in cohort studies assessing the association of the GI with health outcomes. Sixth, the extent to which GI for individual food items predicts glucose responses as part of a mixed meal has been discussed (27), but very frequently it can be predicted from the component carbohydrate moieties. The GI might be most accurate when comparing a low GI food item with a high GI food item, without making any other changes in composition of the meal (35, 36). Last, because there is not always coherence between the GI and insulinemic response, some researchers have used the insulin index as well (36–38). However, the methodology of GI analysis and study design has been steadily improving and issues resolved (27, 33, 34, 39).

The association between the GI and body weight has been covered in a systematic review on macronutrients, food consumption and weight changes, within the NNR project (40).

#### Dietary guidelines for Americans 2010

In the Report of the Dietary Guidelines Advisory Committee on the Dietary Guidelines for Americans, 2010 (13) the chapter on carbohydrates is partly based on systematic reviews. For GI and GL, four scientific questions were asked by the Committee (Table 4).

**Table 4:** Questions asked and the conclusions of the systematic literature reviews for the dietary guidelines for Americans 2010 regarding the GI

Question	Conclusion	Number of studies
What is the relationship between glycemic index or glycemic load and body weight?	‘Strong and consistent evidence shows that glycemic index and/or glycemic load are not associated with body weight and do not lead to greater weight loss or better weight maintenance.’	Based on 22 studies, 13 randomized TC, 2 prospective, 7 cross sectional.
What is the relationship between glycemic index or glycemic load and type 2 diabetes?	‘A moderate body of inconsistent evidence supports a relationship between high glycemic index and type 2 diabetes.’ ‘Strong, convincing evidence shows little association between glycemic load and type 2 diabetes.’	Based on 10 prospective studies. Based on 10 prospective studies.
What is the relationship between glycemic index or glycemic load and cardiovascular disease?	‘Due to limited evidence, no conclusion can be drawn to assess the relationship between either glycemic index or load and cardiovascular disease.’	Based on 8 studies.
What is the relationship between glycemic index or glycemic load and cancer?	‘Abundant, strong epidemiological evidence demonstrates that there is no association between glycemic index or load and cancer.’	Based on 20 prospective longitudinal studies, 2 case cohort and 5 case control studies.

A description of the Nutrition Evidence Library (NEL) evidence-based systematic review process (a web-based state of the art electronic system) can be found in Part C of the report (http://www.cnpp.usda.gov/Publications/DietaryGuidelines/2010/DGAC/Report/C-Methodology.pdf). It was used to address the majority of the science based research questions posed by the Committee. Additional information about the NEL search strategies and criteria used to review each question can be found online at: www.nutritionevidencelibrary.com

The report concludes, ‘When selecting carbohydrate foods, there is no need for concern with their glycemic index or glycemic load. What is important to being mindful of their calories, caloric density, and fiber content.’

#### The scientific opinion of the European Food Safety Authority

The opinion by EFSA gives a review (not a systematic literature review) of studies investigating the GI or GL and glucose tolerance and insulin sensitivity (13 studies), serum lipids (four original studies, and one meta-analysis based on 15 studies), body weight (11 interventions, four cohorts studies and one review), type 2 diabetes (11 studies, one meta-analysis), cardiovascular disease (six studies and one systematic review) and colorectal cancer (meta-analysis based on 11 studies). It concludes that ‘Although there is some support for a role of GI and GL in the treatment of type 2 diabetes and some evidence suggesting that lowering GI and GL may have favorable effects on some metabolic risk factors such as serum lipids, the evidence regarding their role in the prevention of diet-related diseases is still inconclusive.’(6)

In 2011, the same EFSA panel published a scientific opinion on the substantiation of health claims related to reduction of post-prandial glycemic responses for different sugar replacers (xylitol, sorbitol, mannitol, maltitol, lactitol, isomalt, erythritol, D-tagatose, isomaltulose, sucralose, or polydextrose). The opinion concluded that reduction of post-prandial glycemic responses (as long as post-prandial insulinemic responses are not disproportionally increased) may be a beneficial physiological effect, for example to subjects with impaired glucose tolerance, common in the general population of adults.

#### FAO/WHO scientific update on carbohydrates in human nutrition 2007

The previous report from FAO/WHO Expert Consultation published in 1998 suggested that the concept of GI might provide useful means of helping to select the most appropriate carbohydrate containing foods for the maintenance of health and the treatment of several diseases (41). The current update from 2007 does not take as this as clear and points out some of the weaknesses of the GI concept and strongly expresses that a choice of carbohydrate rich foods could not be done on the basis of GI alone. GI is perhaps most appropriately used to guide food choices when considering similar carbohydrate-containing foods, for example bread with a low GI may be preferable to a higher GI bread, with a resultant lower GL, but should always be considered in the context of other nutritional factors. Furthermore, the report points out that although many manufactured food items have been tested for GI, most of the studies which have demonstrated a health benefit of low GI involved the use of naturally occurring and minimally processed foods. They suggest that manufactured foods be further examined, rather than to assume health benefits only on the basis of their functionality, i.e. their glycemic response. The update concludes, however, that despite the reservations made, distinguishing between foods with large enough differences in GI may produce some benefit in terms of glycemic control in diabetes and lipid management. Further, that low dietary GL may reduce the risk of type 2 diabetes and cardiovascular disease, but the same caveats as described above should be used when interpreting such observations.

#### Recommendations by the World Cancer Research Fund and the American Institute for Cancer Research

The systematic review and meta-analysis from the World Cancer Research Fund published in 2010 is an update based on the previous Expert Report published 2007 (42). The updated report found limited evidence for an association between the GI of the diet and cancer risk (breast and colorectal cancer), and no conclusion was drawn (43, 44). However, the report did not elaborate on the total number, type or quality of studies reviewed for their findings.

#### The Tema Nord report: glycemic index – from research to nutrition recommendations?

The Tema Nord report published in 2005 focus on the GI and GL and their associations with metabolic risk factors and diseases as well as overweight/obesity and satiety (9). The report is not a systematic review but includes more than 100 papers. The report concluded that:

For individuals with diabetes or impaired glucose tolerance a low-GI diet might be of importance; this holds as well for those prone to diabetes due to overweight. More evidence is needed to be able to draw more secure conclusions on the importance of low GI food for healthy individuals.

#### Review of studies on GI and the GL from the Nordic countries published in 2000–2011

From the 35 identified abstracts, 12 relevant studies were included. The included studies are summarized in Table 5. One study examined the relation with serum lipids, one study examined cardiovascular disease, one study examined heart failure, three studies examined myocardial infarction (MI) and one study examined type 2 diabetes. The remaining five studies examined the relation with different types of cancers, breast cancer (two studies), stomach cancer (one study), endometrial cancer (one study) and colorectal cancer (one study). All of the papers were prospective cohort studies.

**Table 5:** Nordic studies on GI and GL and different metabolic conditions and diseases

	Outcome	*n*	Sex	Age	Follow up	Adjustments	Results
Simila et al. (45)	Type 2 diabetes	25,943 smokers 1,098 incident diabetes cases	M	50–69 years	12 years	Age, intervention group, BMI, smoking (years and number of cigarettes per day), physical activity, total energy intake, alcohol, energy adjusted intakes of fat and fiber and coffee consumption.	RR for highest vs. lowest quintile for GI was 0.87 (95% CI: 0.71, 1.07) and for GL 0.88 (95% CI: 0.65, 1.17). Substitution of medium GI carbohydrates with high GI: RR for highest vs. lowest quintile 0.75 (95% CI: 0.59, 0.96).
Levitan et al. (46)	Cardiovascular disease	36,246 myocardial infarction (*n*=1,324), ischemic stroke (*n*=692), hemorrhagic stroke (*n*=165) cardiovascular mortality (*n*=785), or all-cause mortality (*n*=2,959)	M	45–79 years	5–8 years	Age, BMI, physical activity, hypertension, smoking, energy and fiber intake.	GL: RR 1.04 (95% CI: 0.80, 1.34), ischemic stroke GL: RR 1.05 (95% CI: 0.74, 1.49), cardiovascular mortality GL: RR 1.13 (95% CI: 0.81, 1.56) or all-cause mortality GL: RR 0.94 (95% CI: 0.79, 1.11). Trend for a greater risk of hemorrhagic stroke RR=1.44 comparing extreme quartiles (95% CI: 0.91, 2.27); *p* for trend=0.047 of dietary GL.
Levitan et al. (47)	Heart failure (HF)	36,019 (639 HF events)	F	48–83 years	9 years	Age, education, BMI, physical activity, smoking, living alone, postmenopausal hormone use, energy, alcohol, fiber, sodium, saturated fat, polyunsaturated fat, protein and carbohydrate intake, family history, hypertension, high cholesterol.	RR for highest vs. lowest quintile for GI was 1.12 (95% CI: 0.87, 1.45; *p* for trend =0.31) and for GL 1.30 (95% CI: 0.87, 1.93; *p* for trend=0.16).
Mursu et al. (56)	Acute myocardial infarction (AMI)	1,981 (376 AMI events)	M	42–60 years	16.1 years	Age, examination year, smoking, BMI, systolic blood pressure, hypertension medication, serum HDL and LDL cholesterol, triglycerides, physical activity, education, family history of cardiovascular disease and diabetes, alcohol and energy intake and energy adjusted intake of folate, fiber, vitamin C, polyunsaturated and saturated fat.	RR for AMI in the highest vs. lowest quartile of GI was 1.25 (95% CI: 0.92, 1.69; *p* for trend=0.08) and for GL 1.11 (95% CI: 0.79, 1.57; *p* for trend=0.21). Among overweight men, RR in the highest compared to the lowest tertile of GI and GL were 1.58 (95% CI: 1.03, 2.43; *p* for trend=0.04, *p* for interaction=0.01) and 2.05 (95% CI: 1.30, 3.23; *p* for trend=0.002, *p* for interaction=0.002), respectively. For physically less active men; energy expenditure for leisure-time physical activity<50 kcal/d, the RR for AMI was 1.72 (95% CI: 1.07–2.76; *p* for trend=0.04, *p* for interaction 0.80) with higher GL.
Levitan et al. (47)	Myocardial infarction (MI)	36,234 (1,138 events)	F	48–83 years	9 years	Age, education, BMI, physical activity, smoking, living alone, postmenopausal hormone, aspirin use, intake of energy, alcohol, fiber, saturated fat, polyunsaturated fat, protein and carbohydrates, family history of myocardial infarction before 60 years, hypertension and high cholesterol.	GI, comparing top to bottom quartile, RR 1.12 (95% CI: 0.92, 1.35; *p*-trend=0.24), GL 1.22 (95% CI: 0.90, 1.65; *p*-trend=0.23).
Jakobsen et al. (48)	Myocardial infarction	53,644 (1,943 events)	M/F	50–62 years	12 years	Age, sex, BMI, education, smoking, physical activity and hypertension, intake of glycemic carbohydrates, proteins, monounsaturated fatty acids, polyunsaturated fatty acids as percentages of total energy intake, energy and alcohol intake.	Replacing saturated fatty acids (SFAs) with carbohydrates with low-GI values is associated with an indicative lower risk of MI (hazard ratio (HR) for MI per 5% increment of energy intake from carbohydrates: 0.88; 95% CI: 0.72, 1.07). Replacing SFAs with carbohydrates with high-GI values is associated with a higher risk of MI (HR: 1.33; 95% CI: 1.08, 1.64).
Oxlund and Heitmann (49)	Serum lipids	335	M/F	35–65 years	6 years	Total cholesterol as endpoint – age, education, BMI, smoking, physical activity, serum triglyceride at baseline, intake of energy, alcohol, fat, carbohydrate and protein and added sugar. LDL as endpoint – age, education, BMI, smoking, physical activity, LDL at baseline, intake of energy, alcohol, added sugar, fiber, systolic blood pressure and coffee.	Dietary GI and GL were related to 6-year changes in serum lipid levels. In men GI was directly related to changes in total cholesterol (DeltaTC), β=0.0044 (95% CI: 0.0008, 0.0081) and GL was positively related to changes in low-density lipoprotein cholesterol (DeltaLDL), β=0.1554 (95% CI: 0.0127, 0.2982).
Larsson et al. (50)	Stomach cancer	61,433 (156 incident cases)	F	40–75 years	17.4 years	Age, education, BMI and intake of energy and alcohol.	Hazard ratios for highest vs. lowest quintile for GI was 0.77 (95% CI: 0.46, 1.30) and for GL 0.76 (95% CI: 0.46, 1.25).
Larsson et al. (51)	Colorectal cancer	61,433 (870 incident cases)	F	40–75 years	17.4 years	Age, date of enrollment, education, BMI, intake of energy, alcohol, cereal fiber, folate, calcium, magnesium and red meat.	Hazard ratios for highest vs. lowest quintile for GI was 1.00 (95% CI: 0.75, 1.33) and for GL 1.06 (95% CI: 0.81, 1.39).
Larsson et al. (52)	Endometrial cancer	61,226 (608 cases)	F	40–75 years	15.6 years	Models stratified by BMI and physical activity adjusted for age. GL adjusted for total energy intake. Models tested for education, age at menarche, oral contraceptive use, age at first birth, parity, age at menopause, postmenopausal hormone use and menopausal status, diabetes, smoking but not included in final analysis.	Rate ratios (RR) for highest vs. lowest quintile for GI was 1.00 (95% CI: 0.77, 1.30) and for GL 1.15 (95% CI: 0.88, 1.51). Among overweight women with low physical activity the RR comparing extreme quartiles was 2.99 (95% CI, 1.17–7.67) for GL.
Larsson et al. (53)	Breast cancer	61,433 women (2,952 incident cases of invasive breast cancer)	F	40–75 years	17.4 years	Age, education, BMI, height, parity, age at first birth, age at menarche, age at menopause, use of oral contraceptives, use of postmenopausal hormones, family history, intake of alcohol, fiber and energy.	Overall RR for highest vs. lowest quintile for GI was 1.08 (95% CI: 0.96, 1.21; *p* for trend=0.20) and for GL 1.13 (95% CI: 1.00, 1.29; *p* for trend=0.05). For estrogen receptor (ER)+/ progesterone receptor (PR)+ RR was 0.89 (95% CI: 0.74, 1.06; *p* for trend=0.32) for GI and 0.94 (95% CI: 0.77, 1.13; *p* for trend=0.59) for GL. For ER-/PR- breast tumors RR was 1.29 (95% CI: 0.85, 1.96; *p* for trend=0.62) for GI and 1.23 (95% CI: 0.79, 1.90; *p* for trend=0.45). For ER+/PR- breast cancer RR 1.44 (95% CI=1.06, 1.97; *p* for trend=0.04) for GI and 1.81 (95% CI=1.29, 2.53; *p* for trend 0.0008) for GL.
Nielsen et al. (54)	Breast cancer incidence	23,870 women (634 incident cases)	F	50–65 years	5–9 years	Parity (parous/nulliparous, number of births and age at first birth, education, use of hormone replacement therapy (HRT), duration of HRT, intake of alcohol and BMI.	Overall incidence rate ratio (IRR) was 0.94 (0.80–1.10) per 10 units per day for GI and 1.04 (0.90–1.19) per 100 units per day for GL. For ER+, IRR was 0.86 (0.71–1.04) per 10 units per day for GI and 0.99 (0.84–1.17) per 100 units per day for GL. For ER-, IRR was 1.46 (1.01–2.11) per 10 units per day for GI and 1.17 (0.86–1.59) per 100 units per day for GL.

M/F: male/female.

No association was found between the estimated GI or GL of the diet and risk for type 2 diabetes (45), ischemic cardiovascular disease (46) or heart failure (55). Out of the three studies on MI, one study did not find an association (47), one study reported a positive association only among obese or physically inactive individuals (56), and the third study reported a lower risk of MI if replacing saturated fatty acids with low GI food instead of high GI food on equal percentage of energy basis (48). The study on serum lipids found an association albeit weak and generally in sub-groups as described before (49). No association was found with stomach cancer (50) or colorectal cancer (51), while a positive association was found between GI and endometrial cancer only among overweight women with low physical activity (52). For breast cancer, one of the two studies (53, 54) found an overall weak positive association with GL (53) while both studies found positive associations with sub-types of breast cancer.

Additionally, four Nordic studies on the GI concept, which did not fit the eligibility criteria, are of interest. Two of the studies focused on ways to change the GI of different food items using resistant starch (chilled potatoes) and vinegar (57, 58), a study finding consumption of barley kernels compared to white wheat bread in the previous evening meal to lower the glucose tolerance when giving the same breakfast to both groups of healthy volunteers (59), and one cross-over study finding lower postprandial glucose and insulin response in healthy subjects when adding 4 g of oat β-glucans to a bread compared with no addition (60). All efforts were made to identify relevant Nordic studies. However, not all researchers mark their studies (index, title or abstract) by country and therefore relevant studies from the Nordic countries might have been missed.

## Conclusions

Based on the current guidelines, scientific reports and the identified papers covering Nordic populations presented in this summary, there is evidence that a high DF intake could protect against development of cardiovascular disease and colorectal cancer. There is moderate evidence that DF is associated with a lower risk of type 2 diabetes. Consumption of foods rich in DF such as fruit, vegetables, whole grain, nuts and legumes should be recommended and further research on different types of DF and their overall health effect are encouraged.

The hypothesis that the postprandial changes in glucose and insulin after a meal might over time trigger different conditions and diseases is important (61–64). However, the findings with health outcomes are inconsistent. The overall conclusion from the Report of the Dietary Guidelines Advisory Committee on the Dietary Guidelines for Americans (2010), the Scientific Opinion from EFSA (2010), FAO/WHO scientific update on carbohydrates in human nutrition (2007) and the Tema Nord report (2005) is that there is suggestive but inconsistent evidence that food with high GI may be associated with an increased risk for type 2 diabetes and cardiovascular disease, especially in those who are overweight or obese, but that evidence is insufficient to incorporate the GI into dietary recommendations for healthy adults. For other health outcomes, the evidence is weaker. The inconsistency in results could partly be explained by methodological issues related to the GI and GL.

Intervention studies, including studies conducted in the Nordic countries, indicate that by changing individual high GI food with a low GI food, such as adding different types of DF to ordinary bread, in the same category without making any other changes may have positive health effects in overweight people or in those with impaired glucose tolerance (35, 58, 64). Some of the cohort studies also suggest that overweight individuals or individuals with abnormal glucose metabolism may especially benefit from a low GI diet and that flexibility in options for dietary counseling based on patient preference could be considered (65).

Nonetheless, it is still unclear how much of the possible health effects are due to the GI per se, and how much additional benefit a low GI diet may offer after compliance with recommendations to increase intake of DF, whole grains, legumes and fruits and vegetables. The physiological effect of meals on glucose and insulin responses is only part of a larger picture of the physiological effect of food on the body after a meal. Therefore, ranking foods solely on acute glucose responses to food might not provide enough information on the overall effects of foods on most common health outcomes. Furthermore, geographic differences might be found in the associations between GI/GL and risk for diseases depending on staple food consumed (61). Further studies should focus on the methodological challenges and to clarify the understanding of the validity and practicality of the GI concept and the potential additional health benefit compared to current recommendations.

## Appendix I

**Table:** Search string used to identify studies on fiber in the Nordic countries against predecided end points

(“Dietary Fiber”[Majr] OR
((“fiber”[TIAB] OR “fibre”[TIAB]) AND diet*[TIAB]))
AND
(“Lipoproteins”[Mesh] OR
“Lipoproteins, HDL”[Mesh] OR
“Lipoproteins, LDL”[Mesh] OR
“Triglycerides”[Mesh] OR
“Triglycerides” [Title/Abstract] OR
“Cholesterol”[Mesh] OR
“Cholesterol” [Title/Abstract] OR
“serum lipids”[Title/Abstract] OR
Low density lipoprotein* [Title/Abstract] OR
High density lipoprotein* [Title/Abstract] OR
LDL [Title/Abstract] OR
HDL [Title/Abstract] OR
“Inflammation Mediators”[Mesh] OR
“Inflammation”[Mesh] OR
“Inflammation”[Title/Abstract] OR
“C-Reactive Protein”[Mesh] OR
“C-reactive protein”[Title/Abstract] OR
“Leukocyte Count”[Mesh] OR
“Hyperglycemia”[Mesh] OR
“Glucose Intolerance”[Mesh] OR
“Blood Glucose”[Mesh] OR
“blood glucose” [Title/Abstract] OR
“impaired fasting glucose”[Title/Abstract] OR
“high fasting glucose”[Title/Abstract] OR
“fasting plasma glucose”[Title/Abstract] OR
“Hemoglobin A”[Mesh] OR
“Hemoglobin A, Glycosylated”[Mesh] OR
“glycosylated”[Title/Abstract] OR
“Insulin Resistance”[Mesh] OR
“insulin resistance”[Title/Abstract] OR
“Hyperinsulinism”[Mesh] OR
“hyperinsulinemia”[Title/Abstract] OR
“insulin sensitivity”[Title/Abstract] OR
“Insulin”[Title/Abstract] OR
“Cardiovascular Diseases”[Mesh] OR
“Cardiovascular disease”[Title/Abstract] OR
“Myocardial Ischemia”[Mesh] OR
“Myocardial Ischemia”[Title/Abstract] OR
“Myocardial Infarction”[Mesh] OR
“Myocardial Infarction”[Title/Abstract] OR
“Stroke”[Mesh] OR
“Stroke” [Title/Abstract] OR
“Coronary Disease”[Mesh] OR
“Coronary Disease”[Title/Abstract] OR
“diabetes”[Title/Abstract] OR
“Diabetes Mellitus”[Mesh] OR
“Diabetes Mellitus, Type 2”[Mesh] OR
“Mortality“[Mesh] OR
“Mortality”[Title/Abstract] OR
“Survival”[Mesh] OR
“Fatal Outcome”[Mesh] OR
“Cause of Death”[Mesh] OR
“Neoplasms”[Mesh] OR
“Cancer”[Title/Abstract])
NOT (“animals”[MeSH Terms:noexp] NOT “humans”[MeSH:noexp])
AND (“2000/01/01”[PDAT]: “2011/12/31”[PDAT])
AND (“Review”[ptyp])
AND (“Scandinavia”[Mesh] OR
“Finland”[Mesh] OR
“Iceland”[Mesh] OR
Scandinavia*[Title/abstract] OR
“Nordic”[Title/abstract] OR
“Sweden”[Title/abstract] OR
“Denmark”[Title/abstract] OR
“Norway”[Title/abstract] OR
“Finland”[Title/abstract] OR
“Iceland”[Title/abstract] OR
“Swedish”[Title/abstract] OR
“Norwegian”[Title/abstract] OR
“Danish”[Title/abstract] OR
“Finnish”[Title/abstract] OR
“Icelandic”[Title/abstract])

## Appendix II

**Table:** Search string used to identify studies on glycemic index and glycemic load in the Nordic countries against predecided end points

(“Glycemic Index”[Mesh] OR
“Glycemic index”[Title/abstract] OR
“glycemic load”[Title/abstract])
AND
(“Lipoproteins”[Mesh] OR
“Lipoproteins, HDL”[Mesh] OR
“Lipoproteins, LDL”[Mesh] OR
“Triglycerides”[Mesh] OR
“Triglycerides”[Title/Abstract] OR
“Cholesterol”[Mesh] OR
“Cholesterol” [Title/Abstract] OR
“serum lipids”[Title/Abstract] OR
Low density lipoprotein*[Title/Abstract] OR
High density lipoprotein*[Title/Abstract] OR
LDL[Title/Abstract] OR
HDL[Title/Abstract] OR
“Inflammation Mediators”[Mesh] OR
“Inflammation”[Mesh] OR
“Inflammation”[Title/Abstract] OR
“C-Reactive Protein”[Mesh] OR
“C-reactive protein”[Title/Abstract] OR
“Leukocyte Count”[Mesh] OR
“Hyperglycemia”[Mesh] OR
“Glucose Intolerance”[Mesh] OR
“Blood Glucose”[Mesh] OR
“blood glucose”[Title/Abstract] OR
“impaired fasting glucose”[Title/Abstract] OR
“high fasting glucose”[Title/Abstract] OR
“fasting plasma glucose”[Title/Abstract] OR
“Hemoglobin A”[Mesh] OR
“Hemoglobin A, Glycosylated”[Mesh] OR
“glycosylated”[Title/Abstract] OR
“Insulin Resistance”[Mesh] OR
“insulin resistance”[Title/Abstract] OR
“Hyperinsulinism”[Mesh] OR
“hyperinsulinemia”[Title/Abstract] OR
“insulin sensitivity”[Title/Abstract] OR
“Insulin”[Title/Abstract] OR
“Cardiovascular Diseases”[Mesh] OR
“Cardiovascular disease”[Title/Abstract] OR
“Myocardial Ischemia”[Mesh] OR
“Myocardial Ischemia”[Title/Abstract] OR
“Myocardial Infarction”[Mesh] OR
“Myocardial Infarction”[Title/Abstract] OR
“Stroke”[Mesh] OR
“Stroke”[Title/Abstract] OR
“Coronary Disease”[Mesh] OR
“Coronary Disease”[Title/Abstract] OR
“diabetes”[Title/Abstract] OR
“Diabetes Mellitus”[Mesh] OR
“Diabetes Mellitus, Type 2”[Mesh] OR
“Mortality“[Mesh] OR
“Mortality”[Title/Abstract] OR
“Survival”[Mesh] OR
“Fatal Outcome”[Mesh] OR
“Cause of Death”[Mesh] OR
“Neoplasms”[Mesh] OR
“Cancer”[Title/Abstract])
**NOT** (“animals”[MeSH Terms:noexp] NOT “humans”[MeSH:noexp])
**AND** (“2000/01/01”[PDAT]: “2011/12/31”[PDAT])
**AND**
(“Scandinavia”[Mesh] OR
“Finland”[Mesh] OR
“Iceland”[Mesh] OR
Scandinavia*[Title/abstract] OR
“Nordic”[Title/abstract] OR
“Sweden”[Title/abstract] OR
“Denmark”[Title/abstract] OR
“Norway”[Title/abstract] OR
“Finland”[Title/abstract] OR
“Iceland”[Title/abstract] OR
“Swedish”[Title/abstract] OR
“Norwegian”[Title/abstract] OR
“Danish”[Title/abstract] OR
“Finnish”[Title/abstract] OR
“Icelandic”[Title/abstract])
